# Supplementary material for: Identification of photoperiod-induced specific miRNAs in the adrenal glands of Sunite sheep (Ovis aries)
Source: Front Vet Sci. 2022 Jul 22;9:888207. doi: 10.3389/fvets.2022.888207 (PMC9354845; doi:10.3389/fvets.2022.888207)
Supplement: Supplementary file 1 [file Data_Sheet_1.ZIP › Supplementary file/miRDeep2 file.zip!/miRDeep2 file/Know_Structure/SP42A1/oar-mir-329b.pdf]

miRBase precursor : oar-mir-329b
Total read count : 1421
oar-miR-329b-5p read count : 13
oar-miR-329b-3p read count : 1402
remaining reads : 6

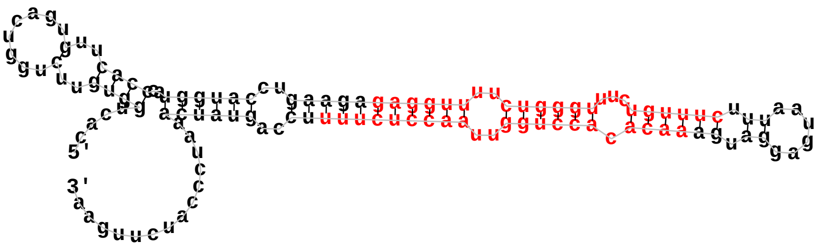

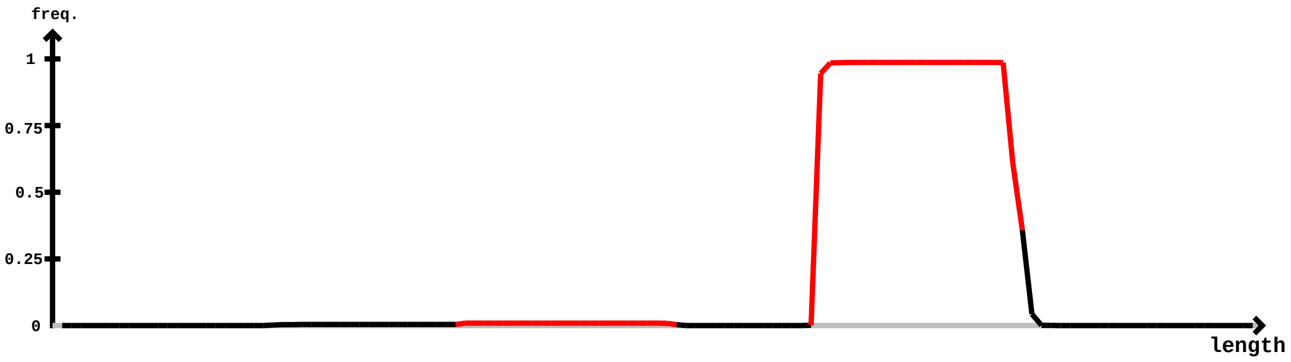

oar-miR-329b-3p

oar-miR-329b-5p

5'- cacugguguucuggucaguguucacccaugguaccugaagagagguuuucuggguuucuguuucuuuaaugaggaugaaacacaccugguuaaccucuuuuccaguaucaaaucccaucuugaa -3'	exp	
....((((..(........)..))))..((((((..(((((((((((..((((((...((((((.((......)).)))))).))))))..)))))))))))..))))))..............	reads	mm
.....................ucacccaugguaccugaaga...	3	0
......................cacccaugguaccugaaga...	2	0
........................cccaugguaccugaaga...	1	0
....gagguuuucuggguuucuguu..	1	0
....gagguuuucuggguuucuguuu...	7	0
....gagguuuucuggguuucuguuuc..	5	0
....aaacacaccugguuaaccucu..........................	2	0
....aacacaccugguuaaccuc...........................	1	0
....aacacaccugguuaaccucu..........................	528	0
....aacacaccugguuaaccucuu.........................	358	0
....aacacaccugguuaaccucuuu........................	432	0
....aacacaccugguuaaccucuuuu.......................	22	0
....acacaccugguuaaccucu..........................	3	0
....acacaccugguuaaccucuu.........................	1	0
....acacaccugguuaaccucuuu........................	15	0
....acacaccugguuaaccucuuuu.......................	37	0
....cacaccugguuaaccucuuu........................	1	0
....cacaccugguuaaccucuuuu.......................	1	0
....acaccugguuaaccucuuuucc.....................	1	0
